# Influence of Filter Pore Size on Composition and Relative Abundance of Bacterial Communities and Select Host-Specific MST Markers in Coastal Waters of Southern Lake Michigan

**DOI:** 10.3389/fmicb.2021.665664

**Published:** 2021-07-15

**Authors:** Muruleedhara N. Byappanahalli, Meredith B. Nevers, Dawn Shively, Cindy H. Nakatsu, Julie L. Kinzelman, Mantha S. Phanikumar

**Affiliations:** ^1^U.S. Geological Survey, Great Lakes Science Center, Chesterton, IN, United States; ^2^Department of Civil and Environmental Engineering, Michigan State University, East Lansing, MI, United States; ^3^Department of Agronomy, Purdue University, West Lafayette, IN, United States; ^4^Public Health Department Laboratory, Racine, WI, United States

**Keywords:** bacterial community composition, 16S rRNA sequencing, filter pore size, filter processing, Great Lakes, host-specific MST markers, sample volume

## Abstract

Water clarity is often the primary guiding factor in determining whether a prefiltration step is needed to increase volumes processed for a range of microbial endpoints. In this study, we evaluate the effect of filter pore size on the bacterial communities detected by 16S rRNA gene sequencing and incidence of two host-specific microbial source tracking (MST) markers in a range of coastal waters from southern Lake Michigan, using two independent data sets collected in 2015 (bacterial communities) and 2016–2017 (MST markers). Water samples were collected from river, shoreline, and offshore areas. For bacterial communities, each sample was filtered through a 5.0-μm filter, followed by filtration through a 0.22-μm filter, resulting in 70 and 143 filter pairs for bacterial communities and MST markers, respectively. Following DNA extraction, the bacterial communities were compared using 16S rRNA gene amplicons of the V3–V4 region sequenced on a MiSeq Illumina platform. Presence of human (*Bacteroides* HF183) and gull (Gull2, *Catellicoccus marimammalium*) host-specific MST markers were detected by qPCR. Actinobacteriota, Bacteroidota, and Proteobacteria, collectively represented 96.9% and 93.9% of the relative proportion of all phyla in the 0.22- and 5.0-μm pore size filters, respectively. There were more families detected in the 5.0-μm pore size filter (368) than the 0.22-μm (228). There were significant differences in the number of taxa between the two filter sizes at all levels of taxonomic classification according to linear discriminant analysis (LDA) effect size (LEfSe) with as many as 986 taxa from both filter sizes at LDA effect sizes greater than 2.0. Overall, the Gull2 marker was found in higher abundance on the 5.0-μm filter than 0.22 μm with the reverse pattern for the HF183 marker. This discrepancy could lead to problems with identifying microbial sources of contamination. Collectively, these results highlight the importance of analyzing pre- and final filters for a wide range of microbial endpoints, including host-specific MST markers routinely used in water quality monitoring programs. Analysis of both filters may increase costs but provides more complete genomic data *via* increased sample volume for characterizing microbial communities in coastal waters.

## Introduction

Significant, recent advancements in the field of molecular biology have contributed to increased application of molecular (i.e., genetic) tools in water quality monitoring programs. Notable examples include the use of polymerase chain reaction (PCR) and quantitative PCR (qPCR) to monitor fecal indicator bacteria and/or human pathogens and to identify contamination sources (Bej et al., 1990; Mahbubani et al., 1991; Scott et al., 2002; Brinkman et al., 2003). More recent advances include high-throughput DNA sequencing (e.g., 16S rRNA amplicon sequencing or similar targets) to better identify and characterize contamination sources and potential interactions among waterways using microbial communities as an index of water quality (McLellan et al., 2009; Newton et al., 2013; Staley and Sadowsky, 2016). These molecular methods and other emerging technologies (e.g., environmental DNA; high-throughput sequencing) are increasingly incorporated into monitoring programs—as a stand-alone method or in conjunction with traditional monitoring methods—with endpoints ranging from measuring shoreline water quality (Wade et al., 2006) to assessing population of fish and other aquatic biota (Goldberg et al., 2016; Lacoursière-Roussel et al., 2016). For water, membrane filtration remains the most common method to concentrate cells and/or DNA from organisms whether the targets are select bacteria, host-specific MST markers, microbial communities, or other biota of interest (e.g., fish and macroinvertebrates) (Venter et al., 2004; Harwood et al., 2014; Goldberg et al., 2016). Selection of filter pore size is largely dependent on water clarity, sample type, and volume as well as characterizing biotic communities by size, composition, and their association with particulate matter (Fuhrman et al., 1988; Venter et al., 2004; Walsh et al., 2009; Francy et al., 2013; Padilla et al., 2015).

Increasingly, a prefiltration step is included as part of sample processing protocols to overcome clogging issues when samples are generally turbid and to ensure that sufficient cells (biota)/DNA are collected from samples by increasing the sample volume for a range of applications (e.g., to differentiate particle-bound and free or unbound cells) (Venter et al., 2004; Staley et al., 2013; Padilla et al., 2015). However, there is little consistency in selecting pore-size filters across water types; often, prevailing water conditions (e.g., dissolved organic matter, turbidity) and measured microbial endpoints (e.g., viruses) (Mull and Hill, 2012; Francy et al., 2013; Xu and Guo, 2017) dictate filter selection. Further, processing of prefilters varies widely across studies: from size-specific, independent extractions to co-extractions (Staley et al., 2013; Padilla et al., 2015; Jain and Krishnan, 2017), which makes study comparisons difficult. Thus, the goal of the current study was to evaluate the influence of filter pore size on composition and relative abundance of bacterial communities and select host-specific microbial source tracking (MST) markers detected in a range of coastal waters along southern Lake Michigan (Wisconsin, Illinois, and Indiana). For this purpose, we used two independent data sets collected over two different time periods: 2015 for 16S rRNA gene sequencing and 2016–2017 for host-specific MST markers to identify human (HF183) and gull (Gull2) fecal contamination.

## Materials and Methods

### Study Area and Sampling Design

The study area is located along the southwestern shoreline of Lake Michigan within the following cities: Racine, Wisconsin; Chicago, Illinois, and East Chicago and Whiting, Indiana. The sampling sites in Racine included the Root River mouth (RRM), an upstream location on the Root River (REC), an offshore location at the marina harbor wall (NBW), and an engineered wetland outlet at North Beach (NB; IEB overflow) as well as a nearshore location at NB, a recreational beach. In Chicago, the 63rd Street Beach was the sole sampling location. In East Chicago, samples were collected from the Grand Calumet River at Columbus Drive (GCR), the mouth of the GCR (GCM), three shoreline locations at Jeorse Park (JP, a recreational beach), and two offshore locations: north (GCN) and east (GCE) of a constructed peninsula; in Whiting, samples were collected from the Whihala Beach (WHW). Additional details of sample collection are available elsewhere (Nakatsu et al., 2019; Kinzelman et al., 2020; Nevers et al., 2020).

Water samples were collected over three summers, 2015–2017. There were 70 water samples collected in 2015 (0.22- and 5.0-μm pore size filters) for 16S rRNA gene sequence analysis and 143 water samples collected in 2016–2017 for MST marker analysis; all water samples were filtered through 0.22- and 5.0-μm pore size filters (see Table 1). Bacterial community data gathered from the 0.22-μm pore size filters have been published elsewhere in two separate studies: 63 filters from Illinois and Indiana locations (Nakatsu et al., 2019) and 54 filters from Wisconsin locations only (Kinzelman et al., 2020). Similarly, the MST data from the 0.22-μm pore size filters (143), except for samples from NB2, NB4, and RRM, are published in a separate study (Nevers et al., 2020). The data related to the 5.0-μm pore size filters—70 (i.e., from 16S rRNA sequencing) and 143 (MST)—are independent and unpublished work. Results from the 0.22-μm pore size filters are used for comparison purposes only and acknowledged appropriately here and elsewhere in this manuscript.

**TABLE 1:** Study locations, sampling years, and the measured microbial endpoints (16S rRNA gene sequencing and two host-specific MST markers) in coastal waters along southern Lake Michigan.

		Analysis		Year		
			
Site	Coordinates (Lat/Long)	16S rRNA gene sequencing	Host-specific MST markers	2015	2016	2017
JP	41.650478/−87.433551	X	X	X	X	X
GCE	41.667120/−87.404940	X		X		
GCN	41.692170/−87.415550	X		X		
GCM	41.687190/−87.439740	X		X		
GCR	41.639400/−87.471276	X	X	X	X	X
WHW	41.685118/−87.492283	X	X	X		
63rd	41.782209/−87.572926	X	X	X	X	X
IEB outlet	42.746607/−87.781631	X		X		
NB	42.741666/−87.779915	X	X	X	X	X
NBW	42.737361/−87.774867	X		X		
RRM	42.733424/−87.771695	X	X	X	X	X
REC	42.724360/−87.795930	X	X	X	X	X

**FIGURE 1:**
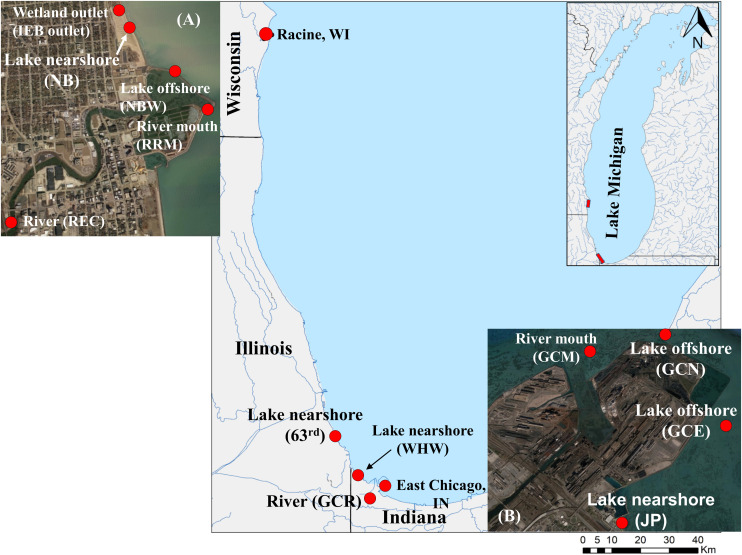
Map of southern Lake Michigan depicting the sampling locations. Water samples were collected from river, shoreline, and offshore sites from Racine, Wisconsin; Chicago, Illinois; and East Chicago, Indiana, locations bordering southern Lake Michigan. The graphical illustration is adapted from Nevers et al. (2020). The sampling sites for river, river mouth, lake near shore, and offshore as well as wetland are shown in the enlarged insets: **(A)** Racine and **(B)** Jeorse Park.

*Water samples were collected (as denoted by symbol X) from Racine, Wisconsin [lake nearshore, NB; lake offshore, NBW, river (Root River), REC; river mouth (Root River Mouth), RPM; and wetland outlet, IEB outlet], Illinois (63rd Street Beach, 63rd); and East Chicago, Indiana (lake nearshore, JP and WHW; lake offshore, GCE and GCN; Grand Calumet River, GCR; and river mouth, GCM) as shown in Figure 1.*

### Sample Collection

In the summer of 2015, water samples (1000 ml) were collected in triplicate during three independent events on the following dates (month/day): Illinois and Indiana: 08/12, 09/01, and 09/21; Wisconsin: 08/03, 08/27, and 09/02. The 11 locations represented five sources: (i) nearshore lake, (ii) offshore lake, (iii) river, (iv) river mouth, and (v) wetland outlet (Figure 1 and Table 1). In 2016, individual water samples (1,000 ml) for filter comparison were collected during three events; and in 2017, individual water samples were collected 1 day per week for 11 weeks (06/08–08/10 from Wisconsin, Illinois, and Indiana locations; see Table 1).

Water from GCR was collected by tossing a sterile collection bucket from the bridge crossing at Columbus Drive, offshore surface water samples (GCM, GCN, and GCE) were collected from a boat by dipping sterile collection bottles (1,000 ml capacity) below the surface, and nearshore samples (JP, WHW, 63rd) were similarly collected by dipping collection bottles (1,000 ml capacity) below the surface in 45-cm-deep water. Nearshore water samples at NB were collected in sterile Whirl-Pak^TM^ bags (Nasco, Fort Atkinson, WI, United States) at a depth of 0.65–0.70 m and approximately 0.3 m below the water surface. Samples from the REC and IEB outlet sites were collected using sterile Whirl-Pak bags and a sampling pole, typically at 10 cm below the surface; samples from RPM and an offshore location (NBW) were collected in sterile Nalgene bottles (1,000 ml capacity) using a sampling line (Kinzelman et al., 2020). All samples were stored on ice directly after collection for return to the laboratory and processed within 6 h of collection.

### DNA Extraction

All water samples (1,000 ml) were first filtered through a 5.0-μm nitrocellulose filter (47 mm, EMD Millipore, Billerica, MA, United States); filtrates were then filtered through 0.22-μm nitrocellulose filter (47 mm, EMD Millipore, Billerica, MA, United States), and both filters were placed in separate DNeasy PowerWater Kit bead tubes (Qiagen, Inc., formerly MOBIO PowerWater) and held at −80°C until processing. DNA was extracted from each filter using a DNeasy PowerWater Kit according to manufacturer’s instructions but with one exception: the final DNA elution was performed twice using 50 μl of DNA elution buffer each time for a final extraction volume of 100 μl. DNA concentrations were measured by fluorometric quantitation using a Qubit^®^ instrument and a High Sensitivity dsDNA HS Assay kit (Qubit, Thermo Fisher Scientific, Waltham, MA, United States). Nucleic acid quality (i.e., 260/280 ratio) was measured with a Nanophotometer^®^ Pearl (Implen Inc., Westlake Village, CA, United States).

### qPCR

DNA extracts from water samples collected during 2016–2017 were analyzed for human (HF183) (Green et al., 2014) and gull (Gull-2) (Lu et al., 2008) markers. The primers and probes used, reaction conditions, and assay controls are explained in greater detail elsewhere (Nevers et al., 2018). qPCR assays for the human and gull MST markers were performed using the Bio-Rad CFX^TM^ Connect Real-time PCR Detection System (Bio-Rad, Hercules, CA, United States) in clear 96-well PCR plates containing 25 μl reaction mixtures. All qPCR assays included appropriate positive and no template controls as well as other quality assurance parameters, such as inhibition controls and extraction, filter, and field blanks to confirm that the assays were working within the conditions defined. For instance, each qPCR assay was tested for inhibition by analyzing diluted and undiluted DNA extracts (randomly chosen, 20%); dilutions consisted of 5× to achieve a concentration range of 2–5 ng total DNA.

DNA extracts were stored at −20°C (short-term, typically <1 week) or −80°C (long-term, up to several months) in case reanalysis was required. For each qPCR assay, amplification efficiency and *R*^2^ were calculated; standard curves for all runs had an *R*^2^ ≥ 0.99 with amplification efficiencies ranging between 92–98% and 92–94% for HF183 and Gull2 assays, respectively. Samples were considered inhibited if Cq values (diluted and undiluted) were dissimilar relative to dilution. Cq is the quantification cycle or the cycle number in which the DNA fluorescence increases above any background fluorescence. The lower limit of quantification (LLOQ) was established by averaging the Cq values obtained from the highest dilutions from standard curves across all qPCR runs in which at least two of the three technical replicates were detected. This resulted in an LLOQ of Cq = 37 for Gull2 and HF183 assays (Nevers et al., 2018), corresponding to an average of 33CN/rx (Gull2) and 18 CN/rx (HF183) (Nevers et al., 2020), which were subsequently used to determine if a sample Cq value falls within the range of quantification (ROQ) and detected but not quantifiable (DNQ). Samples were considered positive if the Cq value was within the ROQ and DNQ; samples were considered negative non-detect (ND) if there was no exponential curve crossing the threshold value before cycle 40.

### Illumina 16S rRNA Gene Sequencing

The 16S rRNA gene sequencing of DNA extracts from water samples is explained in greater detail elsewhere (Nakatsu et al., 2019; Kinzelman et al., 2020). Briefly, PCR amplification of the 16S rRNA gene was performed using V3–V4 region primers (343-forward TAC GGR AGG CAG CAG and 804-reverse CTA CCR GGG TAT CTA ATC C) and ∼10 ng of template DNA. Index tags were added using the step out protocol following the manufacturer’s instructions (Illumina, San Diego, CA, United States) (Gloor et al., 2010). All PCR included no template controls. PCR amplicons were quantified using a Nanodrop 3000 fluorospectrometer after staining with the QuantiFluor dsDNA System (Promega^TM^ Corporation, Madison, WI, United States). Equimolar amounts of amplicons from each sample were combined and sent to the Purdue Genomics core facilities, West Lafayette, IN, United States for 2 × 250 paired end sequencing using a MiSeq Illumina system.

### 16S rRNA Gene Sequence Analysis

One set of river samples (designated by W06 and W123, 0.2- and 5.0-μm pore size filters, respectively) was excluded from sequence analysis because of low reads in W06. Sequences from 140 samples were analyzed using the QIIME 2 pipeline version 2021.2 (Bolyen et al., 2019). Demultiplex sequences were trimmed for quality, denoised, paired ends merged, and sorted into representative amplicon sequence variants (ASVs) using DADA2 (Callahan et al., 2016). Taxonomic assignment to ASVs were made using a classifier trained using the SILVA data set (version 138, 99% OTUs from the V3/V4 16S rRNA gene region) (Yilmaz et al., 2014). For phylogenetic analyses, trees were generated using the multi-alignment program MAFFT (Katoh et al., 2002) and FastTree (Price et al., 2010). Rare ASVs (<0.1% of average reads), unclassified ASVs, and chloroplast reads were filtered out of the data set. Sufficient read coverage of samples was determined by rarefaction analysis as well as estimation of Good’s coverage. All 140 samples were included in diversity analyses and were rarified to the same sampling depth of 6400 reads. Alpha diversity was analyzed using Shannon (richness and evenness), and also, Faith’s phylogenetic diversity (PD) was used for phylogenetic richness and Chao1 for species richness estimates and Pielou’s evenness. Beta diversity analysis included non-phylogenetic metrics Bray–Curtis and Jaccard and phylogenetic metrics weighted and unweighted Unifrac (Lozupone et al., 2011).

### Statistical Analyses

QIIME 2 software was used to determine statistically significant differences between 0.22- and 5.0-μm pore size filters for all alpha and beta diversity metrics tested. All statistical tests were nonparametric for non-normally distributed data. Pairwise Kruskal–Wallis tests were performed for alpha diversity metrics, ANOSIM for non-phylogeny and PERMANOVA (999 permutations) (Anderson, 2001) for phylogeny-associated beta diversity metrics. Also, PERMDISP (Anderson, 2006) was used to determine if dispersion contributed to a difference between the two filter groups. Differential abundances in taxonomic groups between filter groups were determined using analysis of composition of microbiomes (ANCOM) (Mandal et al., 2015) and linear discriminant analysis effect size (LEfSe) (Segata et al., 2011).

## Results and Discussion

### Sequence Information

Illumina sequencing produced a total of 7,399,117, non-chimeric, paired-end reads after quality filtering, denoising, and merging with an average read of 52,850.8 ± 47,221.9 (SD), ranging from 6614 to 311,659 reads from 140 samples (70 samples of each of 0.22- and 5.0-μm pore size filters and a control), representing five major sources: nearshore lake, offshore lake, river, river mouth, and wetland outlet (Figure 1 and Table 1). After filtering out read contaminants, alpha and beta diversity comparisons were made using data rarefied to 6400 reads with an average Good’s coverage of 99.9% (range 99–100%).

### Alpha Diversity

**FIGURE 2:**
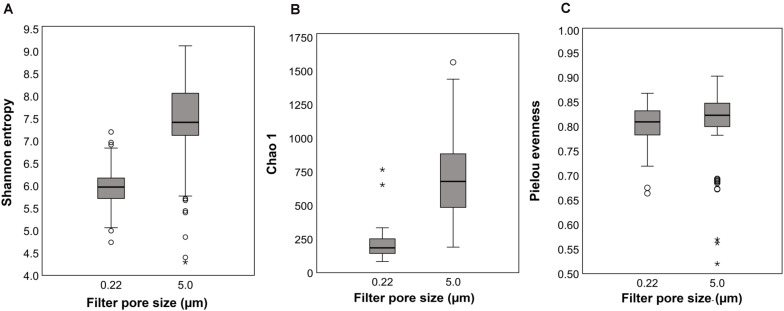
Alpha diversity indices: Boxplots of means and standard deviations (SD) of within-sample diversity from all sampling locations for 0.22- and 5.0-μm pore size filters; **(A)** Shannon (richness and evenness), **(B)** Chao1 richness estimate, and **(C)** Pielou’s community evenness.

Rarefaction curves indicated saturation was reached for Shannon, Pielou’s evenness, and Chao1 indices at read numbers used for statistical comparisons. Shannon index, a measure of richness and evenness, was statistically higher in the 5.0-μm (filter) fraction than the 0.22-μm fraction (H = 58.42, *q* ≤ 0.0001; Figure 2A). Both richness and evenness differences contributed as indicated by differences in Pielou’s community evenness index (H = 5.74, *q* = 0.0165; Figure 2C) and Chao1 richness estimate (H = 93.24, *q* ≤ 0.0001; Figure 2B). These results show that, when multiple filters are used for processing water samples for microbial community analysis (e.g., pre- and final filters in the current study), relying on results from only one filter size (pre- or final filter) underestimates bacterial communities both qualitatively and quantitatively (Mestre et al., 2017). One of the main reasons for adding a prefiltration step in the current study was to increase the sample volume to 1,000 ml for the final filtration through the 0.22-μm pore size filter for 16S rRNA sequencing as reported elsewhere (Nakatsu et al., 2019; Kinzelman et al., 2020). Analysis of the 5.0-μm filters (current study) provided an opportunity to compare results between the two filter fractions and to characterize the observed differences at different taxonomic levels. Further, portioning the bacterial communities into free-living or attached to particulate matter was not an objective of this study; however, the higher community richness in the 5.0-μm filter fraction might be attributed to bacteria most likely associated with plankton and other particulate matter (e.g., soil, sediments) collected on the larger pore size filter (Padilla et al., 2015; Mestre et al., 2017; Borrego et al., 2020).

### Beta Diversity

**FIGURE 3:**
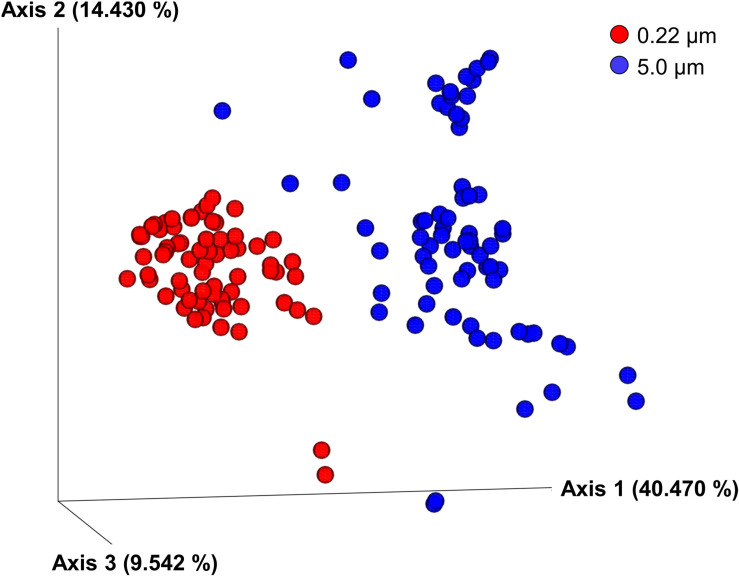
Principal coordinate analysis (PCoA) of weighted Unifrac metrics of samples labeled according to filter pore size (i.e., 0.22- and 5.0-μm filters). Differences among bacterial communities were significant according to PERMANOVA and ANOSIM tests (*q* = 0.001).

Clear differences between communities of the 5.0- and 0.22-μm fractions were seen in PCoA plots using all the beta diversity indices tested (results are shown only for weighted Unifrac; Figure 3). The main difference seen was in PCoA axis 1, accounting for 40.47% of the variation for weighted Unifrac; collectively, the three axes accounted for more than 64% of the variation. Both PERMANOVA and ANOSIM indicated these differences were significant for all metrics (*q* = 0.001). Despite the significant differences in PERMDISP, the PCoA plot shows that the communities do not overlap along PCoA1. The dispersion of communities was greater in communities from the 5.0-μm fraction compared with the 0.22-μm fractions along PCoA2, indicating greater variability of communities in the 5.0 μm fraction. A variety of environmental factors (e.g., temperature, season, and water stratifications) (Cram et al., 2015; Sunagawa et al., 2015; Cheng et al., 2020) are known to contribute to differences in observed beta diversity. Filter pore size is another factor that can affect the beta diversity, especially when the bacterial communities in water samples include free-living and particle-associated fractions; in this scenario, the particle-associated communities are likely to be retained on the larger pore size filter (i.e., 5.0 μm in the current study) (Padilla et al., 2015; Mestre et al., 2017) as previously discussed. Additionally, bacterial size, shape, and flexibility are known to affect their passage through membrane filters (Wang et al., 2008), resulting in differential distribution of communities between the two pore size filters used in this study.

### Taxonomic Representation

#### Phyla

**FIGURE 4:**
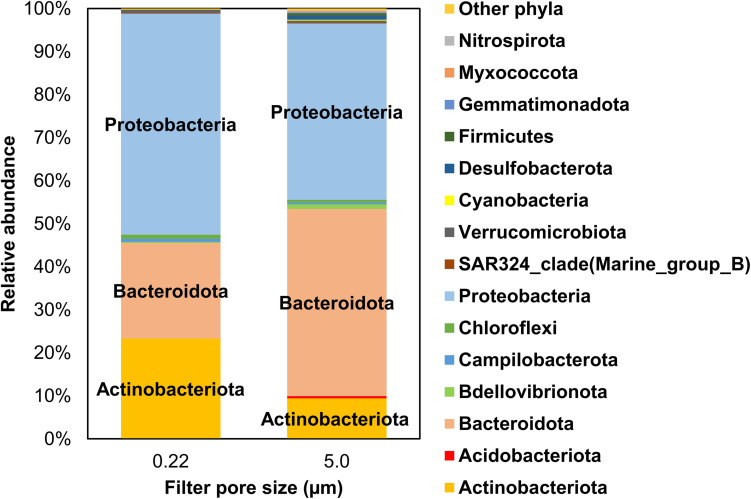
Mean relative abundance of bacterial phyla identified in water samples from all sampling locations by filter pore size (i.e., 0.22- and 5.0-μm filters). Only phyla >0.1% by relative abundance are included in this illustration.

Overall, Actinobacteriota, Bacteroidota (formerly, Bacteroidetes), and Proteobacteria collectively represented 96.9% and 93.9% (by relative proportion of all phyla) in 0.22- and 5.0-μm pore size filters, respectively (Figure 4). Individually, comparing 0.22- and 5.0-μm pore size filters, Actinobacteriota were 23.3% ± 8.24% (mean and SD) vs. 9.4% ± 5.2%, Bacteroidota 22.2% ± 5.1% vs. 43.5% ± 11.5%, and Proteobacteria 51.3% ± 6.4% vs. 41.0% ± 8.9%. Although the dominance of these phyla is consistent with our recent studies in Lake Michigan (Nakatsu et al., 2019; Kinzelman et al., 2020) and other similar studies elsewhere (Newton et al., 2011; Mou et al., 2013), their differential relative abundances between the two filter fractions are noteworthy. Because these phyla are equally abundant in soil (Janssen, 2006) and aquatic vegetation (e.g., algae) (Miranda et al., 2013; Chun et al., 2017), their relative abundances in coastal waters may reflect the possibility of more than one source of these bacteria in interconnecting riparian (streams, rivers) and shoreline waters (Figure 1).

Other phyla with mean relative abundances ≥0.1% included Bdellovibrionota (previously Bdellovibrio), Campilobacterota, and Verrucomicrobiota (all 0.22 μm) and Acidobacteriota, Chloroflexi, Cyanobacteria, Desulfobacterota, and Firmicutes (5.0 μm), among others (Figure 4). In general, 5.0 μm filter fraction had more taxa than the 0.22 μm fraction. Cyanobacteria represented only a fraction of the sequences in both filter fractions (0.04% ± 0.05% and 0.25% ± 0.39% in 0.22 μm and 5.0 μm filters, respectively). Even though the two filter fractions were primarily dominated by Actinobacteriota, Bacteroidota, and Proteobacteria, albeit in different relative abundances, a differential distribution of several less abundant phyla was evident between the two filters. Notably, Cyanobacteria, Desulfobacterota, Firmicutes, Gemmatimonadota, Myxococcota, and Nitrospirota were mainly found in the 5.0-μm fraction, implying that analyzing both filters were required to capture the relative abundance of the measured (identified) taxa in this study.

#### Families

**FIGURE 5:**
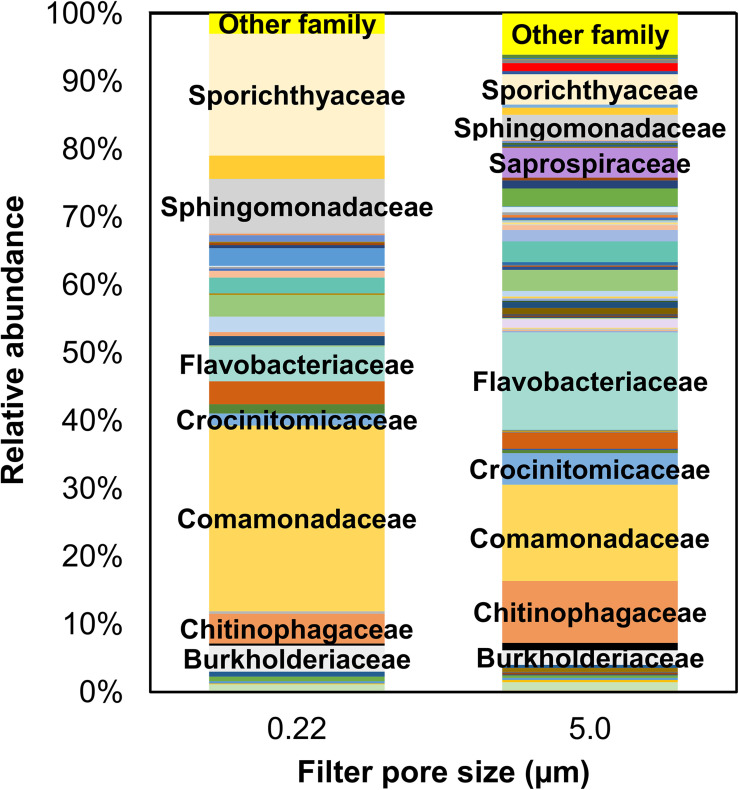
Mean relative abundance of bacterial families identified in water samples from all sampling locations by filter pore size (i.e., 0.22- and 5.0-μm filters). Only families >0.1% by relative abundance are included in this illustration; the rest (<0.1% by relative abundance) are included under “Others.”

Like phyla, there were more families identified in the 5.0-μm pore size filter (368) than 0.22-μm (228). Only data with relative abundances >0.1% are graphically depicted in Figure 5; families with <0.1% in relative abundance are included under “other.” Several families were common in both 0.22- and 5.0-μm filter fractions, albeit in varying proportions: *Burkholderiaceae* (3.8% vs. 2.1%), *Chitinophagaceae* (4.3% vs. 9.1%), *Comamonadaceae* (27.3% vs. 14.2%), *Crocinitomicaceae* (1.7% vs. 4.6%), *Flavobacteriaceae* (5.1% vs. 14.7%), *Sphingomonadaceae* (8.1% vs. 3.8%), and *Sporichthyaceae* (18.0% vs. 4.5%).

Interestingly, families comprising the traditional fecal indicators, such as *Enterobacteriaceae* (*E. coli*), *Enterococcaceae* (*Enterococcus* sp.), *Bacteroidaceae* (*Bacteroides* sp.), *Clostridiaceae* (*Clostridium*, especially *C. perfringens*), and *Lachnospiraceae* (Harwood et al., 2014), had sequence reads either too low (i.e., <0.1 by relative abundance)—*Bacteroidaceae, Enterobacteriaceae*, and *Lachnospiraceae*—or not detected in the samples (*Enterococcaceae* and *Clostridiaceae*) in both filter fractions. Thus, the 16S rRNA sequencing method might not be sensitive enough to detect these indicators in certain situations due to low sequence reads. However, any comparison of results related to the bacterial communities (noted above) between the two methods evaluated in this study (i.e., 16S rRNA gene sequencing and MST markers by qPCR) is tenuous because the data were not collected at the same time and qPCR detection is more sensitive than 16S rRNA sequencing.

#### Taxa Differences Between Filter Fractions

**FIGURE 6:**
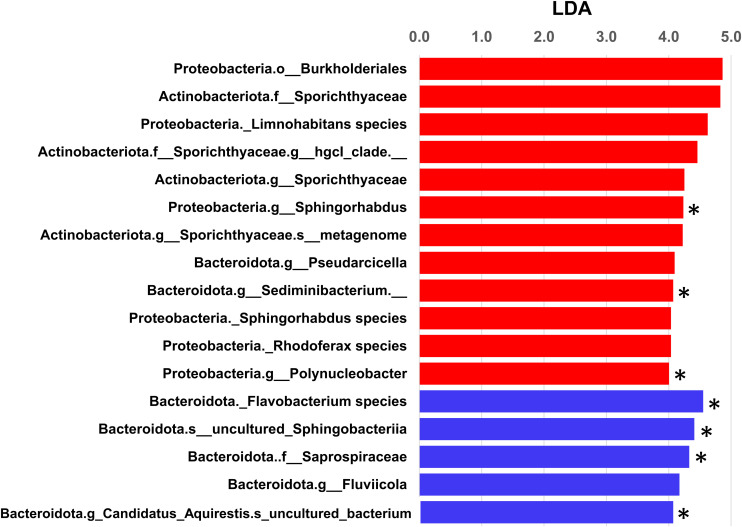
Linear discriminate analysis effect size (LEfSe) analysis of bacterial communities from the two filter fractions analyzed (i.e., 0.22- and 5.0-μm filters). Taxa differed significantly at all levels of taxonomic classification. Only taxa that could be assigned to the genus level with LDA effect sizes >4.0 are shown in this graphical illustration (red and blue bars represent observed taxa in 0.22- and 5.0-μm filters, respectively; ^∗^ denotes taxa that were significant by ANCOM). Overall, there were 986 taxa with LDA effect sizes greater than 2.0 (Supplementary Table 1).

Linear discriminant analysis effect size analysis identified significant differences in taxa between the two filter fractions at all levels of taxonomic classification. There were 986 taxa with linear discriminant analysis (LDA) effect sizes greater than 2.0 (Supplementary Table 1). Those that could be classified to the genus level with the highest LDA effect sizes (>4.0, Figure 6) included *Limnohabitans*, *Sporichthyaceae* hgcl clade, *Sphigorhabdus*, *Pseudarcicella*, *Sediminibacterium, Rhodoferax*, and *Polynucleobacter* in the 0.22-μm fraction and *Flavobacterium, Fluviicola*, and Candidatus *Aquirestis* in the 5.0-μm fraction. Seven of these taxa were also significantly different using ANCOM. None of the taxa in either fraction represented bacteria of relevance to traditional indicators of water quality. In previous studies from the same study locations, there were several taxa that differed significantly among water sources (river, river mouth, stormwater wetland, lake near shore, and lake offshore) and collection sites (nearshore and river mouth); however, only 0.22-μm filters were used in those studies (Nakatsu et al., 2019; Kinzelman et al., 2020).

Collectively, noticeable differences were evident between the two pore size filters across all taxa (phyla, family, and genus) evaluated in this study. Such differences might be attributed to (a) cells and/or their DNA attaching to smaller particulate matter, such as clay minerals and organic fractions (Cai et al., 2006; Cuadros, 2018; Harrison et al., 2019; Sirois and Buckley, 2019), and passing through the larger pore size prefilter and (b) potentially unbound (i.e., free) bacterial cells primarily captured by the smaller pore size filters used (0.22 μm in the current study). Previous studies show that bacterial cells (e.g., *Escherichia coli, Hylemonella gracilis, Klebsiella pneumoniae, Pseudomonas aeruginosa*, and *Serratia marcescens*), can pass through pore size filters ranging from 0.1 to 0.45 μm (Hasegawa et al., 2003; Wang et al., 2007).

### Host-Specific MST Markers

In general, the Gull2 marker was found in higher frequency in the 5.0-μm filter than the 0.22-μm filter (71% vs. 61%); however, the pattern was opposite for the HF183 marker: 31% vs. 48% (Table 2). These two markers were found in all water types, except for the upstream river samples from 0.22-μm filters (0/26). A low incidence of the Gull2 marker (12%, 3/26; 5.0-μm filters) indicates that contamination of rivers from gull feces is minimal or sporadic—most likely attributed to urban runoff from parking lots or other sources adjacent to streams (Green et al., 2019). Conversely, a relatively higher frequency of this marker in the river mouth samples (in both 0.22- and 5.0-μm filters from RRM), suggests higher activity of shoreline birds at public parks or recreational areas adjacent to beaches (Whitman and Nevers, 2003; Byappanahalli et al., 2015).

**TABLE 2:** Detection rates of Gull2 and HF183 host-specific MST markers in samples by filter pore size (0.22- and 5.0-μm) and water source [% detected includes samples within range of quantification (ROQ) and detected but not quantifiable (DNQ)].

Source	Percent detected (N)			
	
	Gull2		HF183	
		
	0.22 μm	5.0 μm	0.22 μm	5.0 μm
Overall	61 (87/143)	71 (102/143)	48 (69/143)	31 (45/143)
River	0 (0/26)	12 (3/26)	73 (19/26)	73 (19/26)
River mouth	39 (5/13)	69 (9/13)	77 (10/13)	46 (6/13)
Shoreline	79 (82/104)	87 (90/104)	39 (40/104)	19 (20/104)

The two MST markers evaluated in this study represent two different fecal sources of distinct origin, namely wastewater (HF183) and environmental or wildlife (Gull2). Both markers were detected in both 0.22- and 5.0-μm filters but at different frequencies. One explanation might be that the bacteria (*C. marimammalium*) and/or DNA from gull feces is localized; likely associated with fecal droppings and/or deposited into the sand/sediment matrix underneath because a significantly higher incidence of the Gull2 marker was previously detected in sand and sediment than water at the same study locations (Nevers et al., 2020). Thus, particle-bound cells/DNA from gull feces are likely to get trapped (in the larger pore size prefilter), and the unbound cells/DNA pass through the prefilter. The HF183 marker (from Bacteroidota), on the other hand, primarily comes from human fecal sources (e.g., treated sewage), which undergoes rigorous processing (e.g., screening, agitation or churning, and sedimentation) in wastewater treatment plants, leaving a portion of cells and/or their DNA unbound or attached to much smaller particulate matter such as clays and colloidal particles (Cai et al., 2006; Cuadros, 2018). Further, fecal contamination, originating from failed infrastructure or septic fields, which contributes to overall abundance (HF183 and other Bacteroidetes markers), more likely to be dispersed in the receiving water bodies vs. concentrated cells/DNA on particulate matter (e.g., MST markers in sand in the case of direct deposition from gulls) (Sauer et al., 2011; Nevers et al., 2020). Nonetheless, additional research is needed to better understand the reasons for differential detection of host-specific MST markers between pore size filters, including those not tested in this study as well as exploring other combinations of filter sizes for capturing capacities. In addition, future research should target samples from the same events for multiple analyses because any comparison of results, such as 16S rRNA gene sequencing and MST markers (current study), is tenuous.

## Summary and Conclusion

DNA-based methods are increasingly applied in a wide range of environmental programs: from monitoring shoreline water quality to the assessment of biotic communities in aquatic systems. Membrane filtration remains a foundational step in the processing of water *via* a wide range of molecular assays employed for the identification of microbial or species-specific DNA. A prefiltration step is often included to overcome clogging and to ensure that sufficient cells/DNA are collected for the measured endpoints; however, there is little consistency in processing the pre- and final filters. In the current study, we analyzed the pre- and final filters independently to evaluate their influence on composition and abundance of bacterial communities and two MST markers routinely used in water quality monitoring programs.

Collectively, our findings show that (a) there were both qualitative and quantitative differences in bacterial communities between the two filter sizes with significant differences in number of taxa at all levels of taxonomic representation and (b) the two MST markers, Gull2 and HF183, showed different detection rates between the two filter sizes. Such differential detection could potentially result in an inaccurate or underrepresented pollution source profile when selectively analyzing a single pore size filter (either pre- or the final filter).

In summary, analyzing both pre- and final filters increases our confidence in the results. The molecular target and environmental substrate from which DNA is extracted play a role in the successful recovery and estimation of relative abundance. Targets extracted from hard substrates, such as sand, or highly turbid waters may be more difficult to recover due their adherence to particulate matter (e.g., sediments, clays), which can foul filters employed in the extraction process. When conducting investigative studies, it is important that the full complement of organisms be represented to render the greatest insight. Although analysis of multiple filters may increase costs, it provides more complete genomic data *via* increased sample volume for characterizing microbial communities in coastal waters.

## Data Availability Statement

Nucleotide sequences related to the results from 0.22- and 0.5-mm pore size filters are available in the National Center for Biotechnology Information-Sequence Read Archive (NCBISRA) database under study PRJNA725142 accession numbers SAMN18870789–SAMN18870931 (https://www.ncbi.nlm.nih.gov/bioproject/?term=PRJNA725142). Data generated during this study are available as a USGS data release: Byappanahalli et al. (2021).

## Author Contributions

MB and MN contributed to conception and design of the study, and acquired funding for this research. MB, MN, DS, JK, and CN designed the experiments and conducted the research. CN and DS performed the statistical analyses. MB provided supervision and wrote the first draft. CN, MN, DS, JK, and MP reviewed the first draft. All authors contributed to manuscript revision, read, and approved the submitted version.

## Conflict of Interest

The authors declare that the research was conducted in the absence of any commercial or financial relationships that could be construed as a potential conflict of interest.

## Supplementary Material

Supplementary Figure 1 Volcano plot of ANCOM results, *y*-axis *W*-statistic, and *x*-axis centered log ratio (clr).

Supplementary Table 1 LEfSe results showing taxa differences between 0.22- and 5.0-μm pore size filters at all taxa levels with LDA > 2.0.
